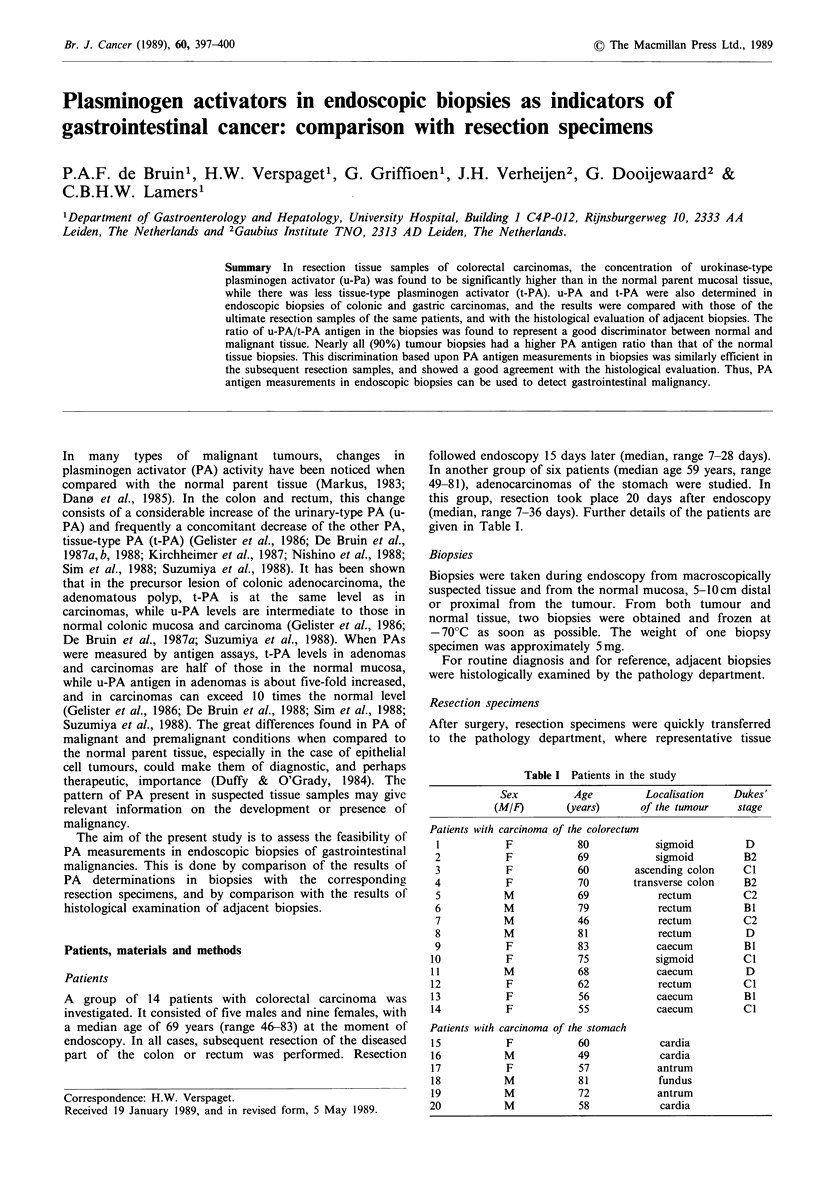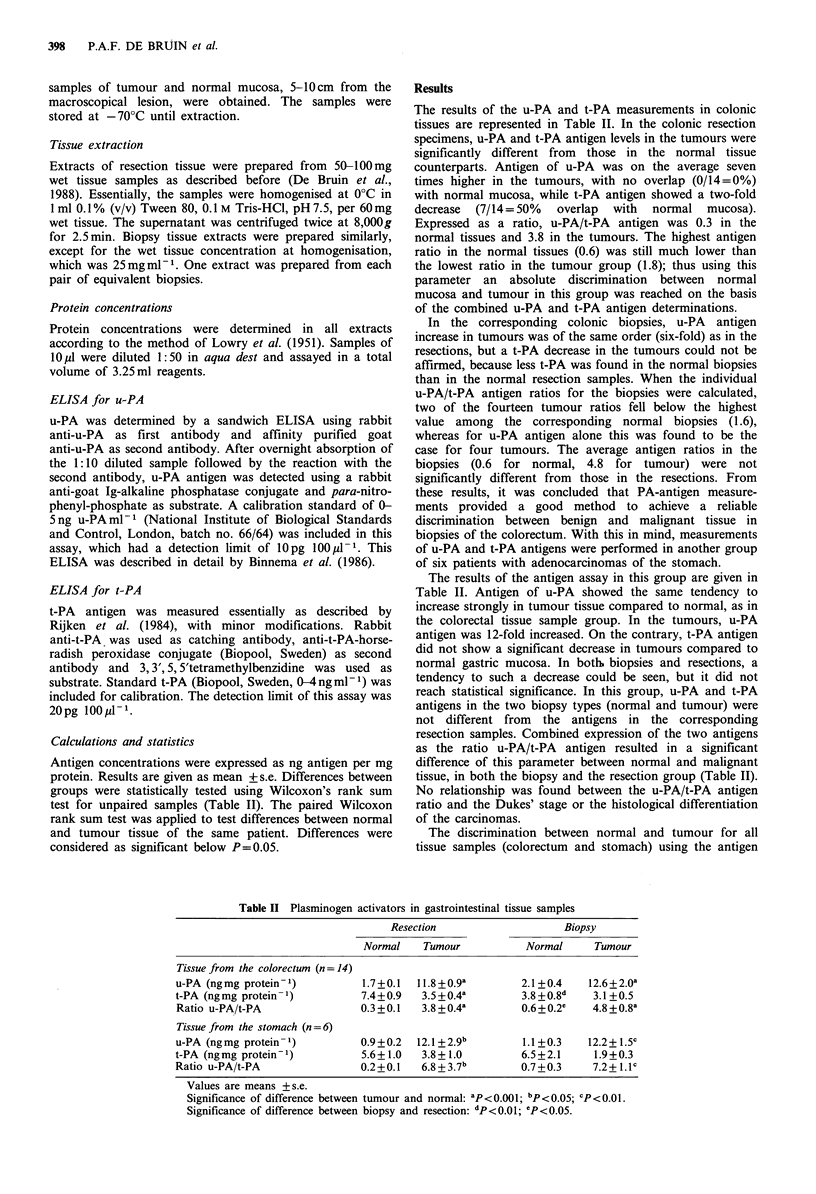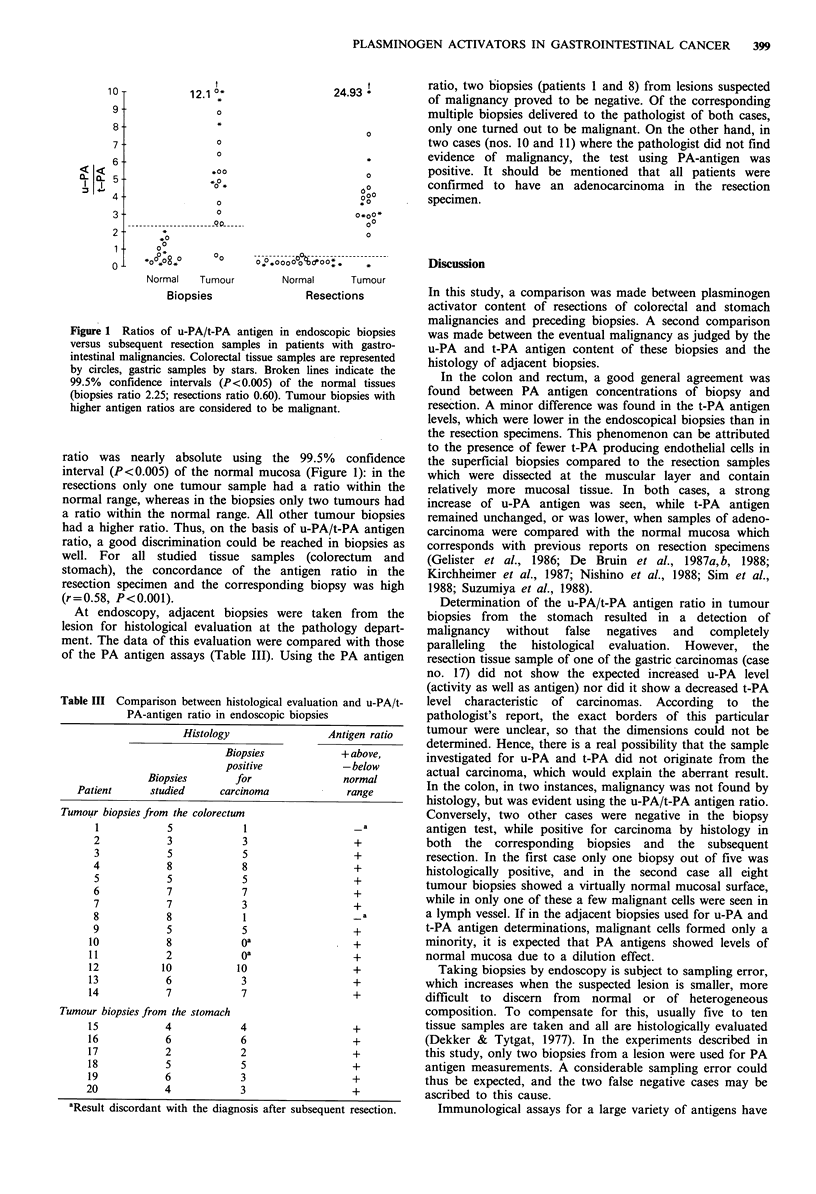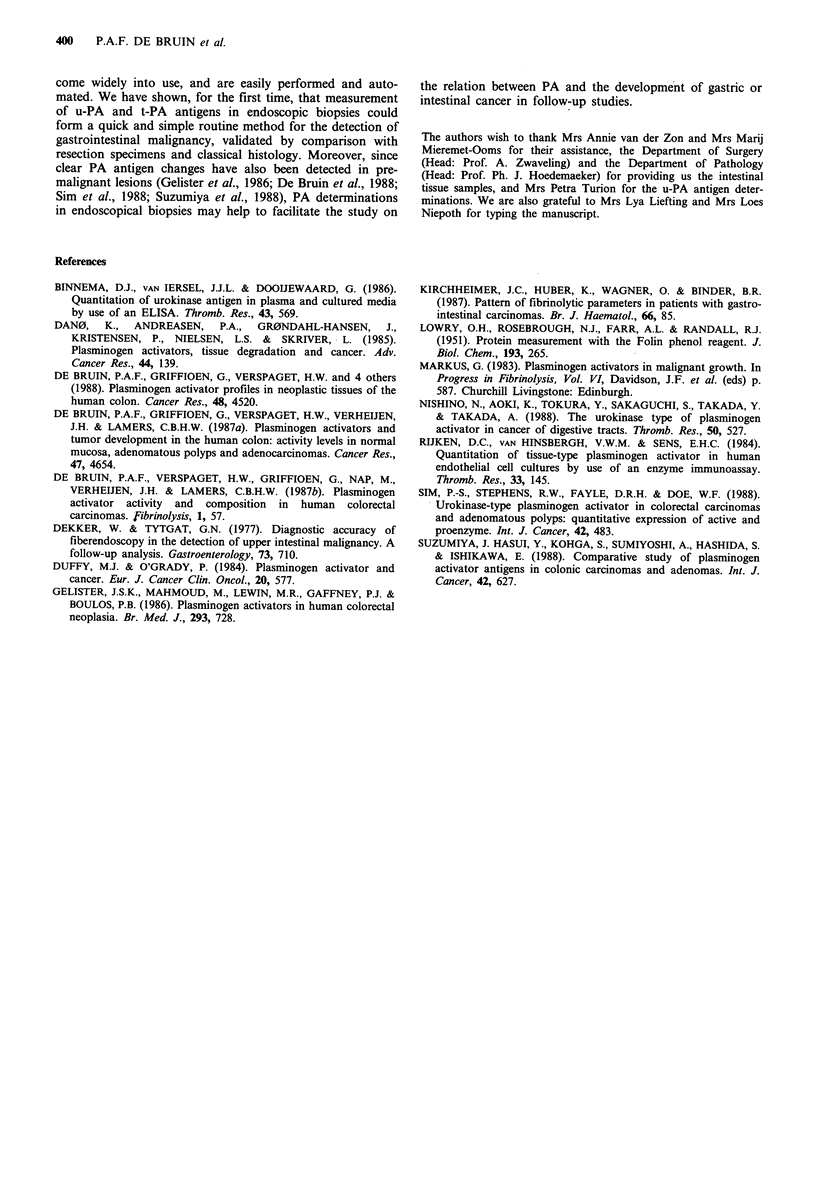# Plasminogen activators in endoscopic biopsies as indicators of gastrointestinal cancer: comparison with resection specimens.

**DOI:** 10.1038/bjc.1989.293

**Published:** 1989-09

**Authors:** P. A. de Bruin, H. W. Verspaget, G. Griffioen, J. H. Verheijen, G. Dooijewaard, C. B. Lamers

**Affiliations:** Department of Gastroenterology and Hepatology, University Hospital, Leiden, The Netherlands.

## Abstract

In resection tissue samples of colorectal carcinomas, the concentration of urokinase-type plasminogen activator (u-Pa) was found to be significantly higher than in the normal parent mucosal tissue, while there was less tissue-type plasminogen activator (t-PA). u-PA and t-PA were also determined in endoscopic biopsies of colonic and gastric carcinomas, and the results were compared with those of the ultimate resection samples of the same patients, and with the histological evaluation of adjacent biopsies. The ratio of u-PA/t-PA antigen in the biopsies was found to represent a good discriminator between normal and malignant tissue. Nearly all (90%) tumour biopsies had a higher PA antigen ratio than that of the normal tissue biopsies. This discrimination based upon PA antigen measurements in biopsies was similarly efficient in the subsequent resection samples, and showed a good agreement with the histological evaluation. Thus, PA antigen measurements in endoscopic biopsies can be used to detect gastrointestinal malignancy.


Br. J. Cancer (1989), 60, 397–400 © The Macmillan Press Ltd., 1989

### Plasminogen activators in endoscopic biopsies as indicators of gastrointestinal cancer: comparison with resection specimens

P.A.F. de Bruin[1], H.W. Verspaget[1], G. Griffioen[1], J.H. Verheijen[2], G. Dooijewaard[2] & C.B.H.W. Lamers[1]

[1]*Department of Gastroenterology and Hepatology, University Hospital, Building 1 C4P-012, Rijnsburgerweg 10, 2333 AA Leiden, The Netherlands and* [2]*Gaubius Institute TNO, 2313 AD Leiden, The Netherlands.*

**Summary** In resection tissue samples of colorectal carcinomas, the concentration of urokinase-type plasminogen activator (u-Pa) was found to be significantly higher than in the normal parent mucosal tissue, while there was less tissue-type plasminogen activator (t-PA). u-PA and t-PA were also determined in endoscopic biopsies of colonic and gastric carcinomas, and the results were compared with those of the ultimate resection samples of the same patients, and with the histological evaluation of adjacent biopsies. The ratio of u-PA/t-PA antigen in the biopsies was found to represent a good discriminator between normal and malignant tissue. Nearly all (90%) tumour biopsies had a higher PA antigen ratio than that of the normal tissue biopsies. This discrimination based upon PA antigen measurements in biopsies was similarly efficient in the subsequent resection samples, and showed a good agreement with the histological evaluation. Thus, PA antigen measurements in endoscopic biopsies can be used to detect gastrointestinal malignancy.

In many types of malignant tumours, changes in plasminogen activator (PA) activity have been noticed when compared with the normal parent tissue (Markus, 1983; Danø *et al.*, 1985). In the colon and rectum, this change consists of a considerable increase of the urinary-type PA (u-PA) and frequently a concomitant decrease of the other PA, tissue-type PA (t-PA) (Gelister *et al.*, 1986; De Bruin *et al.*, 1987*a*,*b*, 1988; Kirchheimer *et al.*, 1987; Nishino *et al.*, 1988; Sim *et al.*, 1988; Suzumiya *et al.*, 1988). It has been shown that in the precursor lesion of colonic adenocarcinoma, the adenomatous polyp, t-PA is at the same level as in carcinomas, while u-PA levels are intermediate to those in normal colonic mucosa and carcinoma (Gelister *et al.*, 1986; De Bruin *et al.*, 1987*a*; Suzumiya *et al.*, 1988). When PAs were measured by antigen assays, t-PA levels in adenomas and carcinomas are half of those in the normal mucosa, while u-PA antigen in adenomas is about five-fold increased, and in carcinomas can exceed 10 times the normal level (Gelister *et al.*, 1986; De Bruin *et al.*, 1988; Sim *et al.*, 1988; Suzumiya *et al.*, 1988). The great differences found in PA of malignant and premalignant conditions when compared to the normal parent tissue, especially in the case of epithelial cell tumours, could make them of diagnostic, and perhaps therapeutic, importance (Duffy & O'Grady, 1984). The pattern of PA present in suspected tissue samples may give relevant information on the development or presence of malignancy.

The aim of the present study is to assess the feasibility of PA measurements in endoscopic biopsies of gastrointestinal malignancies. This is done by comparison of the results of PA determinations in biopsies with the corresponding resection specimens, and by comparison with the results of histological examination of adjacent biopsies.

### Patients, materials and methods

### *Patients*

A group of 14 patients with colorectal carcinoma was investigated. It consisted of five males and nine females, with a median age of 69 years (range 46–83) at the moment of endoscopy. In all cases, subsequent resection of the diseased part of the colon or rectum was performed. Resection followed endoscopy 15 days later (median, range 7–28 days). In another group of six patients (median age 59 years, range 49–81), adenocarcinomas of the stomach were studied. In this group, resection took place 20 days after endoscopy (median, range 7–36 days). Further details of the patients are given in Table I.

### *Biopsies*

Biopsies were taken during endoscopy from macroscopically suspected tissue and from the normal mucosa, 5–10 cm distal or proximal from the tumour. From both tumour and normal tissue, two biopsies were obtained and frozen at −70°C as soon as possible. The weight of one biopsy specimen was approximately 5 mg.

For routine diagnosis and for reference, adjacent biopsies were histologically examined by the pathology department.

### *Resection specimens*

After surgery, resection specimens were quickly transferred to the pathology department, where representative tissue

**Table I** Patients in the study

| | *Sex (M/F)* | *Age (years)* | *Localisation of the tumour* | *Dukes' stage* |
|---|---|---|---|---|
| *Patients with carcinoma of the colorectum* | | | | |
| 1 | F | 80 | sigmoid | D |
| 2 | F | 69 | sigmoid | B2 |
| 3 | F | 60 | ascending colon | C1 |
| 4 | F | 70 | transverse colon | B2 |
| 5 | M | 69 | rectum | C2 |
| 6 | M | 79 | rectum | B1 |
| 7 | M | 46 | rectum | C2 |
| 8 | M | 81 | rectum | D |
| 9 | F | 83 | caecum | B1 |
| 10 | F | 75 | sigmoid | C1 |
| 11 | M | 68 | caecum | D |
| 12 | F | 62 | rectum | C1 |
| 13 | F | 56 | caecum | B1 |
| 14 | F | 55 | caecum | C1 |
| *Patients with carcinoma of the stomach* | | | | |
| 15 | F | 60 | cardia | |
| 16 | M | 49 | cardia | |
| 17 | F | 57 | antrum | |
| 18 | M | 81 | fundus | |
| 19 | M | 72 | antrum | |
| 20 | M | 58 | cardia | |

Correspondence: H.W. Verspaget.
Received 19 January 1989, and in revised form, 5 May 1989.

samples of tumour and normal mucosa, 5–10 cm from the macroscopical lesion, were obtained. The samples were stored at −70°C until extraction.

*Tissue extraction*

Extracts of resection tissue were prepared from 50–100 mg wet tissue samples as described before (De Bruin *et al.*, 1988). Essentially, the samples were homogenised at 0°C in 1 ml 0.1% (v/v) Tween 80, 0.1 M Tris-HCl, pH 7.5, per 60 mg wet tissue. The supernatant was centrifuged twice at 8,000*g* for 2.5 min. Biopsy tissue extracts were prepared similarly, except for the wet tissue concentration at homogenisation, which was 25 mg ml$^{-1}$. One extract was prepared from each pair of equivalent biopsies.

*Protein concentrations*

Protein concentrations were determined in all extracts according to the method of Lowry *et al.* (1951). Samples of 10 $\mu$l were diluted 1:50 in *aqua dest* and assayed in a total volume of 3.25 ml reagents.

*ELISA for u-PA*

u-PA was determined by a sandwich ELISA using rabbit anti-u-PA as first antibody and affinity purified goat anti-u-PA as second antibody. After overnight absorption of the 1:10 diluted sample followed by the reaction with the second antibody, u-PA antigen was detected using a rabbit anti-goat Ig-alkaline phosphatase conjugate and *para*-nitrophenyl-phosphate as substrate. A calibration standard of 0–5 ng u-PA ml$^{-1}$ (National Institute of Biological Standards and Control, London, batch no. 66/64) was included in this assay, which had a detection limit of 10 pg 100 $\mu$l$^{-1}$. This ELISA was described in detail by Binnema *et al.* (1986).

*ELISA for t-PA*

t-PA antigen was measured essentially as described by Rijken *et al.* (1984), with minor modifications. Rabbit anti-t-PA was used as catching antibody, anti-t-PA-horseradish peroxidase conjugate (Biopool, Sweden) as second antibody and 3,3′,5,5′tetramethylbenzidine was used as substrate. Standard t-PA (Biopool, Sweden, 0–4 ng ml$^{-1}$) was included for calibration. The detection limit of this assay was 20 pg 100 $\mu$l$^{-1}$.

*Calculations and statistics*

Antigen concentrations were expressed as ng antigen per mg protein. Results are given as mean ±s.e. Differences between groups were statistically tested using Wilcoxon's rank sum test for unpaired samples (Table II). The paired Wilcoxon rank sum test was applied to test differences between normal and tumour tissue of the same patient. Differences were considered as significant below $P = 0.05$.

**Results**

The results of the u-PA and t-PA measurements in colonic tissues are represented in Table II. In the colonic resection specimens, u-PA and t-PA antigen levels in the tumours were significantly different from those in the normal tissue counterparts. Antigen of u-PA was on the average seven times higher in the tumours, with no overlap (0/14 = 0%) with normal mucosa, while t-PA antigen showed a two-fold decrease (7/14 = 50% overlap with normal mucosa). Expressed as a ratio, u-PA/t-PA antigen was 0.3 in the normal tissues and 3.8 in the tumours. The highest antigen ratio in the normal tissues (0.6) was still much lower than the lowest ratio in the tumour group (1.8); thus using this parameter an absolute discrimination between normal mucosa and tumour in this group was reached on the basis of the combined u-PA and t-PA antigen determinations.

In the corresponding colonic biopsies, u-PA antigen increase in tumours was of the same order (six-fold) as in the resections, but a t-PA decrease in the tumours could not be affirmed, because less t-PA was found in the normal biopsies than in the normal resection samples. When the individual u-PA/t-PA antigen ratios for the biopsies were calculated, two of the fourteen tumour ratios fell below the highest value among the corresponding normal biopsies (1.6), whereas for u-PA antigen alone this was found to be the case for four tumours. The average antigen ratios in the biopsies (0.6 for normal, 4.8 for tumour) were not significantly different from those in the resections. From these results, it was concluded that PA-antigen measurements provided a good method to achieve a reliable discrimination between benign and malignant tissue in biopsies of the colorectum. With this in mind, measurements of u-PA and t-PA antigens were performed in another group of six patients with adenocarcinomas of the stomach.

The results of the antigen assay in this group are given in Table II. Antigen of u-PA showed the same tendency to increase strongly in tumour tissue compared to normal, as in the colorectal tissue sample group. In the tumours, u-PA antigen was 12-fold increased. On the contrary, t-PA antigen did not show a significant decrease in tumours compared to normal gastric mucosa. In both biopsies and resections, a tendency to such a decrease could be seen, but it did not reach statistical significance. In this group, u-PA and t-PA antigens in the two biopsy types (normal and tumour) were not different from the antigens in the corresponding resection samples. Combined expression of the two antigens as the ratio u-PA/t-PA antigen resulted in a significant difference of this parameter between normal and malignant tissue, in both the biopsy and the resection group (Table II). No relationship was found between the u-PA/t-PA antigen ratio and the Dukes' stage or the histological differentiation of the carcinomas.

The discrimination between normal and tumour for all tissue samples (colorectum and stomach) using the antigen

**Table II** Plasminogen activators in gastrointestinal tissue samples

| | *Resection* | | *Biopsy* | |
|---|---|---|---|---|
| | *Normal* | *Tumour* | *Normal* | *Tumour* |
| *Tissue from the colorectum* ($n = 14$) | | | | |
| u-PA (ng mg protein$^{-1}$) | 1.7±0.1 | 11.8±0.9$^a$ | 2.1±0.4 | 12.6±2.0$^a$ |
| t-PA (ng mg protein$^{-1}$) | 7.4±0.9 | 3.5±0.4$^a$ | 3.8±0.8$^d$ | 3.1±0.5 |
| Ratio u-PA/t-PA | 0.3±0.1 | 3.8±0.4$^a$ | 0.6±0.2$^e$ | 4.8±0.8$^a$ |
| *Tissue from the stomach* ($n = 6$) | | | | |
| u-PA (ng mg protein$^{-1}$) | 0.9±0.2 | 12.1±2.9$^b$ | 1.1±0.3 | 12.2±1.5$^c$ |
| t-PA (ng mg protein$^{-1}$) | 5.6±1.0 | 3.8±1.0 | 6.5±2.1 | 1.9±0.3 |
| Ratio u-PA/t-PA | 0.2±0.1 | 6.8±3.7$^b$ | 0.7±0.3 | 7.2±1.1$^c$ |

Values are means ±s.e.
Significance of difference between tumour and normal: $^aP<0.001$; $^bP<0.05$; $^cP<0.01$.
Significance of difference between biopsy and resection: $^dP<0.01$; $^eP<0.05$.

**Figure 1** Ratios of u-PA/t-PA antigen in endoscopic biopsies versus subsequent resection samples in patients with gastro-intestinal malignancies. Colorectal tissue samples are represented by circles, gastric samples by stars. Broken lines indicate the 99.5% confidence intervals ($P<0.005$) of the normal tissues (biopsies ratio 2.25; resections ratio 0.60). Tumour biopsies with higher antigen ratios are considered to be malignant.

ratio was nearly absolute using the 99.5% confidence interval ($P<0.005$) of the normal mucosa (Figure 1): in the resections only one tumour sample had a ratio within the normal range, whereas in the biopsies only two tumours had a ratio within the normal range. All other tumour biopsies had a higher ratio. Thus, on the basis of u-PA/t-PA antigen ratio, a good discrimination could be reached in biopsies as well. For all studied tissue samples (colorectum and stomach), the concordance of the antigen ratio in the resection specimen and the corresponding biopsy was high ($r=0.58$, $P<0.001$).

At endoscopy, adjacent biopsies were taken from the lesion for histological evaluation at the pathology department. The data of this evaluation were compared with those of the PA antigen assays (Table III). Using the PA antigen

**Table III** Comparison between histological evaluation and u-PA/t-PA-antigen ratio in endoscopic biopsies

| | *Histology* | | *Antigen ratio* |
|---|---|---|---|
| *Patient* | *Biopsies studied* | *Biopsies positive for carcinoma* | *+above, −below normal range* |
| *Tumour biopsies from the colorectum* | | | |
| 1 | 5 | 1 | −[a] |
| 2 | 3 | 3 | + |
| 3 | 5 | 5 | + |
| 4 | 8 | 8 | + |
| 5 | 5 | 5 | + |
| 6 | 7 | 7 | + |
| 7 | 7 | 3 | + |
| 8 | 8 | 1 | −[a] |
| 9 | 5 | 5 | + |
| 10 | 8 | 0[a] | + |
| 11 | 2 | 0[a] | + |
| 12 | 10 | 10 | + |
| 13 | 6 | 3 | + |
| 14 | 7 | 7 | + |
| *Tumour biopsies from the stomach* | | | |
| 15 | 4 | 4 | + |
| 16 | 6 | 6 | + |
| 17 | 2 | 2 | + |
| 18 | 5 | 5 | + |
| 19 | 6 | 3 | + |
| 20 | 4 | 3 | + |

[a]Result discordant with the diagnosis after subsequent resection.

ratio, two biopsies (patients 1 and 8) from lesions suspected of malignancy proved to be negative. Of the corresponding multiple biopsies delivered to the pathologist of both cases, only one turned out to be malignant. On the other hand, in two cases (nos. 10 and 11) where the pathologist did not find evidence of malignancy, the test using PA-antigen was positive. It should be mentioned that all patients were confirmed to have an adenocarcinoma in the resection specimen.

### Discussion

In this study, a comparison was made between plasminogen activator content of resections of colorectal and stomach malignancies and preceding biopsies. A second comparison was made between the eventual malignancy as judged by the u-PA and t-PA antigen content of these biopsies and the histology of adjacent biopsies.

In the colon and rectum, a good general agreement was found between PA antigen concentrations of biopsy and resection. A minor difference was found in the t-PA antigen levels, which were lower in the endoscopical biopsies than in the resection specimens. This phenomenon can be attributed to the presence of fewer t-PA producing endothelial cells in the superficial biopsies compared to the resection samples which were dissected at the muscular layer and contain relatively more mucosal tissue. In both cases, a strong increase of u-PA antigen was seen, while t-PA antigen remained unchanged, or was lower, when samples of adeno-carcinoma were compared with the normal mucosa which corresponds with previous reports on resection specimens (Gelister *et al.*, 1986; De Bruin *et al.*, 1987*a*,*b*, 1988; Kirchheimer *et al.*, 1987; Nishino *et al.*, 1988; Sim *et al.*, 1988; Suzumiya *et al.*, 1988).

Determination of the u-PA/t-PA antigen ratio in tumour biopsies from the stomach resulted in a detection of malignancy without false negatives and completely paralleling the histological evaluation. However, the resection tissue sample of one of the gastric carcinomas (case no. 17) did not show the expected increased u-PA level (activity as well as antigen) nor did it show a decreased t-PA level characteristic of carcinomas. According to the pathologist's report, the exact borders of this particular tumour were unclear, so that the dimensions could not be determined. Hence, there is a real possibility that the sample investigated for u-PA and t-PA did not originate from the actual carcinoma, which would explain the aberrant result. In the colon, in two instances, malignancy was not found by histology, but was evident using the u-PA/t-PA antigen ratio. Conversely, two other cases were negative in the biopsy antigen test, while positive for carcinoma by histology in both the corresponding biopsies and the subsequent resection. In the first case only one biopsy out of five was histologically positive, and in the second case all eight tumour biopsies showed a virtually normal mucosal surface, while in only one of these a few malignant cells were seen in a lymph vessel. If in the adjacent biopsies used for u-PA and t-PA antigen determinations, malignant cells formed only a minority, it is expected that PA antigens showed levels of normal mucosa due to a dilution effect.

Taking biopsies by endoscopy is subject to sampling error, which increases when the suspected lesion is smaller, more difficult to discern from normal or of heterogeneous composition. To compensate for this, usually five to ten tissue samples are taken and all are histologically evaluated (Dekker & Tytgat, 1977). In the experiments described in this study, only two biopsies from a lesion were used for PA antigen measurements. A considerable sampling error could thus be expected, and the two false negative cases may be ascribed to this cause.

Immunological assays for a large variety of antigens have come widely into use, and are easily performed and automated. We have shown, for the first time, that measurement of u-PA and t-PA antigens in endoscopic biopsies could form a quick and simple routine method for the detection of gastrointestinal malignancy, validated by comparison with resection specimens and classical histology. Moreover, since clear PA antigen changes have also been detected in premalignant lesions (Gelister *et al.*, 1986; De Bruin *et al.*, 1988; Sim *et al.*, 1988; Suzumiya *et al.*, 1988), PA determinations in endoscopical biopsies may help to facilitate the study on the relation between PA and the development of gastric or intestinal cancer in follow-up studies.

The authors wish to thank Mrs Annie van der Zon and Mrs Marij Mieremet-Ooms for their assistance, the Department of Surgery (Head: Prof. A. Zwaveling) and the Department of Pathology (Head: Prof. Ph. J. Hoedemaeker) for providing us the intestinal tissue samples, and Mrs Petra Turion for the u-PA antigen determinations. We are also grateful to Mrs Lya Liefting and Mrs Loes Niepoth for typing the manuscript.

### References

BINNEMA, D.J., VAN IERSEL, J.J.L. & DOOIJEWAARD, G. (1986). Quantitation of urokinase antigen in plasma and cultured media by use of an ELISA. *Thromb. Res.*, **43**, 569.

DANØ, K., ANDREASEN, P.A., GRØNDAHL-HANSEN, J., KRISTENSEN, P., NIELSEN, L.S. & SKRIVER, L. (1985). Plasminogen activators, tissue degradation and cancer. *Adv. Cancer Res.*, **44**, 139.

DE BRUIN, P.A.F., GRIFFIOEN, G., VERSPAGET, H.W. and 4 others (1988). Plasminogen activator profiles in neoplastic tissues of the human colon. *Cancer Res.*, **48**, 4520.

DE BRUIN, P.A.F., GRIFFIOEN, G., VERSPAGET, H.W., VERHEIJEN, J.H. & LAMERS, C.B.H.W. (1987*a*). Plasminogen activators and tumor development in the human colon: activity levels in normal mucosa, adenomatous polyps and adenocarcinomas. *Cancer Res.*, **47**, 4654.

DE BRUIN, P.A.F., VERSPAGET, H.W., GRIFFIOEN, G., NAP, M., VERHEIJEN, J.H. & LAMERS, C.B.H.W. (1987*b*). Plasminogen activator activity and composition in human colorectal carcinomas. *Fibrinolysis*, **1**, 57.

DEKKER, W. & TYTGAT, G.N. (1977). Diagnostic accuracy of fiberendoscopy in the detection of upper intestinal malignancy. A follow-up analysis. *Gastroenterology*, **73**, 710.

DUFFY, M.J. & O'GRADY, P. (1984). Plasminogen activator and cancer. *Eur. J. Cancer Clin. Oncol.*, **20**, 577.

GELISTER, J.S.K., MAHMOUD, M., LEWIN, M.R., GAFFNEY, P.J. & BOULOS, P.B. (1986). Plasminogen activators in human colorectal neoplasia. *Br. Med. J.*, **293**, 728.

KIRCHHEIMER, J.C., HUBER, K., WAGNER, O. & BINDER, B.R. (1987). Pattern of fibrinolytic parameters in patients with gastrointestinal carcinomas. *Br. J. Haematol.*, **66**, 85.

LOWRY, O.H., ROSEBROUGH, N.J., FARR, A.L. & RANDALL, R.J. (1951). Protein measurement with the Folin phenol reagent. *J. Biol. Chem.*, **193**, 265.

MARKUS, G. (1983). Plasminogen activators in malignant growth. In *Progress in Fibrinolysis, Vol. VI*, Davidson, J.F. *et al.* (eds) p. 587. Churchill Livingstone: Edinburgh.

NISHINO, N., AOKI, K., TOKURA, Y., SAKAGUCHI, S., TAKADA, Y. & TAKADA, A. (1988). The urokinase type of plasminogen activator in cancer of digestive tracts. *Thromb. Res.*, **50**, 527.

RIJKEN, D.C., VAN HINSBERGH, V.W.M. & SENS, E.H.C. (1984). Quantitation of tissue-type plasminogen activator in human endothelial cell cultures by use of an enzyme immunoassay. *Thromb. Res.*, **33**, 145.

SIM, P.-S., STEPHENS, R.W., FAYLE, D.R.H. & DOE, W.F. (1988). Urokinase-type plasminogen activator in colorectal carcinomas and adenomatous polyps: quantitative expression of active and proenzyme. *Int. J. Cancer*, **42**, 483.

SUZUMIYA, J. HASUI, Y., KOHGA, S., SUMIYOSHI, A., HASHIDA, S. & ISHIKAWA, E. (1988). Comparative study of plasminogen activator antigens in colonic carcinomas and adenomas. *Int. J. Cancer*, **42**, 627.